# Immunophenotyping in BK Virus Allograft Nephropathy Distinct from Acute Rejection

**DOI:** 10.1155/2013/412902

**Published:** 2013-09-30

**Authors:** Xue Li, Qiquan Sun, Jinsong Chen, Shuming Ji, Jiqiu Wen, Dongrei Cheng, Zhihong Liu

**Affiliations:** ^1^Research Institute of Nephrology, Jinling Hospital, Nanjing University Clinical School of Medicine, 305 East Zhong Shan Road, Nanjing 210002, China; ^2^Department of Renal Transplantation, The Third Affiliated Hospital, Sun Yat-Sen University, Guangzhou 510760, China

## Abstract

Differentiating BK virus nephropathy (BKVN) from acute rejection (AR) is crucial in clinical practice, as both of them have interstitial inflammation in the grafts. The purpose of the study is to describe the inflammatory cellular constituents of BKVN and to determine the clinical utility of immunophenotyping findings in distinguishing BKVN from AR. In addition, the expression of the HLA-DR was investigated. Sixty-five renal allograft recipients were included in this study, including 22 cases of BKVN, 31 cases of AR, and 12 cases of stable allograft. Immunostaining for infiltrating lymphocytes showed that the number of CD20 cells (*P* < 0.001) and the percentages of CD3 (*P* < 0.001), CD4 (*P* = 0.004), CD8 (*P* = 0.005), and CD20 (*P* = 0.002) cells were all significantly different between BKVN and AR. Moreover, there were no statistically significant differences in tubule cell HLA-DR expression (*P* = 0.156). This observation suggests that the number of CD20 cells and the percentages of CD3, CD4, CD8, and CD20 cells in renal biopsies would aid the distinction between BKVN and AR. On the other hand, the presence of HLA-DR upregulation may not only be specific for acute rejection but also be a response to BKVN.

## 1. Introduction

BK virus nephropathy (BKVN) has been recognized as a cause of renal dysfunction following kidney transplantation. The incidence of biopsy-proven BKVN can vary between 1% and 10% with subsequent graft loss in more than 50% of cases [1–4], which is much worse than other common conditions, such as acute rejection (AR) and calcineurin inhibitor (CNI) toxicity [5]. A definitive diagnosis of BKVN is made by histological examination of a graft biopsy, and the BKVN seen in renal transplant recipients including acute tubular injury and necrosis can mimic AR by light microscopic examination [6, 7]. Early detection of overimmunosuppression may be of value in avoiding the development of BKVN. However, the clinical management of BKVN and AR is completely opposite. Therefore, it is essential to carefully distinguish BKVN from AR. Immunophenotyping has gained widespread consideration for diagnosis and management of viral infections in immunocompromised hosts [8–11]. In a previous study, Ahuja et al. indicated that the predominance of CD20-positive lymphocytes in renal histology is suggestive of BK virus (BKV) infection [12]. They suggested, however, that this aspect marks an opportunity for future research. In this case series, we retrospectively reviewed the histological features of all renal allograft biopsies in kidney transplant recipients diagnosed with BKVN between 2007 and 2013, a period that preceded routine BKV surveillance and the availability of immunophenotyping for infiltrating lymphocytes in renal histology. The purpose of the study is to describe the inflammatory cellular constituents of BKVN and to determine the clinical utility of immunophenotyping findings in distinguishing BKVN from AR.

## 2. Materials and Methods

### 2.1. Patient Selection

**Figure 1:**
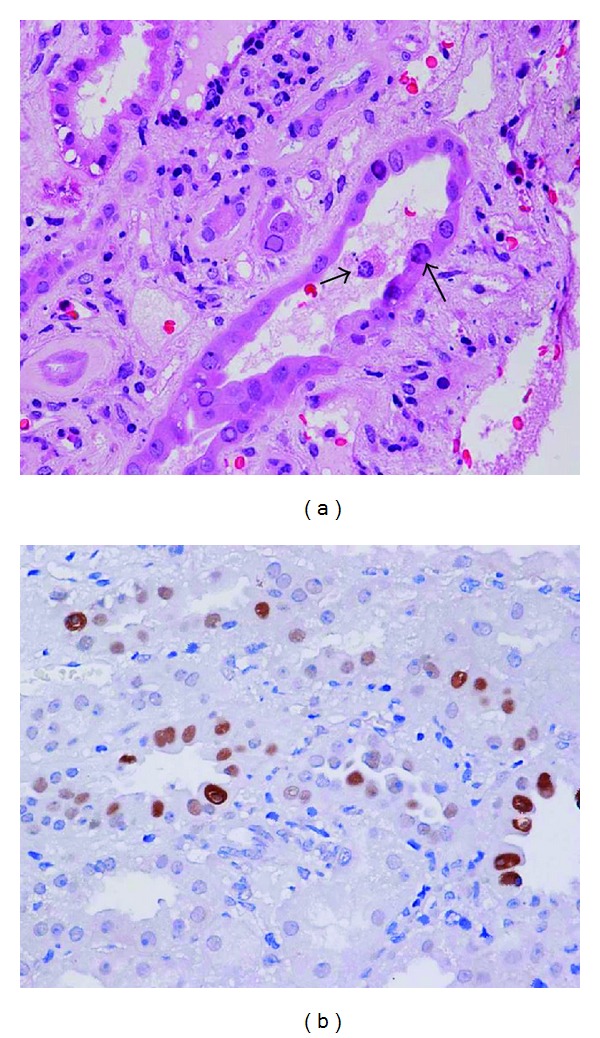
(a) BK virus allograft nephropathy. The histological manifestations are characterized by intranuclear viral inclusions in tubular epithelial cells and epithelial cell necrosis (arrow). Hematoxylin and eosin stained paraffin section. (b) Immunohistochemical staining of a renal biopsy showing positive staining for the BK T antigen.

The patients were retrospectively selected from among 356 renal allograft recipients who had recieved renal biopsy between June 2007 and February 2013 at Jinling Hospital, Nanjing University School of Medicine, Nanjing, China. Informed consent was obtained from all patients, and the Human Subjects Committee of Jinling Hospital, Nanjing University School of Medicine, approved all of the study protocols. Among them, 22 recipients were assigned to the BKVN when the biopsy demonstrated viral cytopathic changes with intranuclear inclusion bodies, associated renal tubular epithelial cell injury including tubular epithelial cell necrosis and denudation of basement membranes, and positive immunohistochemical staining for the BK T antigen (Figure 1). These patients were compared to 31 recipients diagnosed as acute rejection based on the following: (1) clinical evidence of acute rejection, manifested as rapid renal dysfunction and/or decrease of urine volume; (2) pathologic features that met the Banff 97 criteria [13] for acute rejection grade I, II, or III; and (3) without other demonstrable pathology and lacked viral cytopathic changes and T antigen immunostaining. The findings in these two groups were also compared to a group of biopsies from recipients with stable allograft function (SF; *n* = 12), defined as having a protocol biopsy at least 2 weeks after transplantation without a change in serum creatinine (<10% above baseline) and in the absence of any histologic abnormality including drug toxicity, infection, or acute or chronic rejection.

### 2.2. Renal Biopsies

Biopsies were performed upon clinical indication and according to local standard of practice. Two needle biopsy cores were obtained from each renal allograft for morphologic study: one for formalin fixation and the other for quick-freezing. Hematoxylin and eosin, periodic acid Schiff, methenamine-silver, and Masson stains were routinely used on the formalin-fixed tissue. The residual biopsy tissues were stored for future use. Fresh-frozen tissues were analyzed by immunofluorescence microscopy using a conventional panel of antibodies against IgG, IgM, IgA, C3, C4, C1q, HLA-DR, BKV, and C4d. BKV infection was evaluated using anti-BKV antibody (mouse monoclonal antibody specific for anti-BK virus large T antigen; Millipore Biosciences, Temecula, CA, USA). C4d staining was routinely performed on frozen slides using an indirect immunofluorescence technique with a primary affinity-purified monoclonal antibody (mouse anti-human; Quidel, San Diego, CA) and an FITC-labeled affinity-purified secondary rabbit anti-mouse IgG antibody (Dako, Denmark). The staining was performed using standard procedures. Positive C4d staining was defined as a bright linear stain along the capillary basement membranes that involved over half of the sampled capillaries in accordance with the 2001 Banff Meeting [14].

### 2.3. Immunohistological Analysis

**Figure 2:**
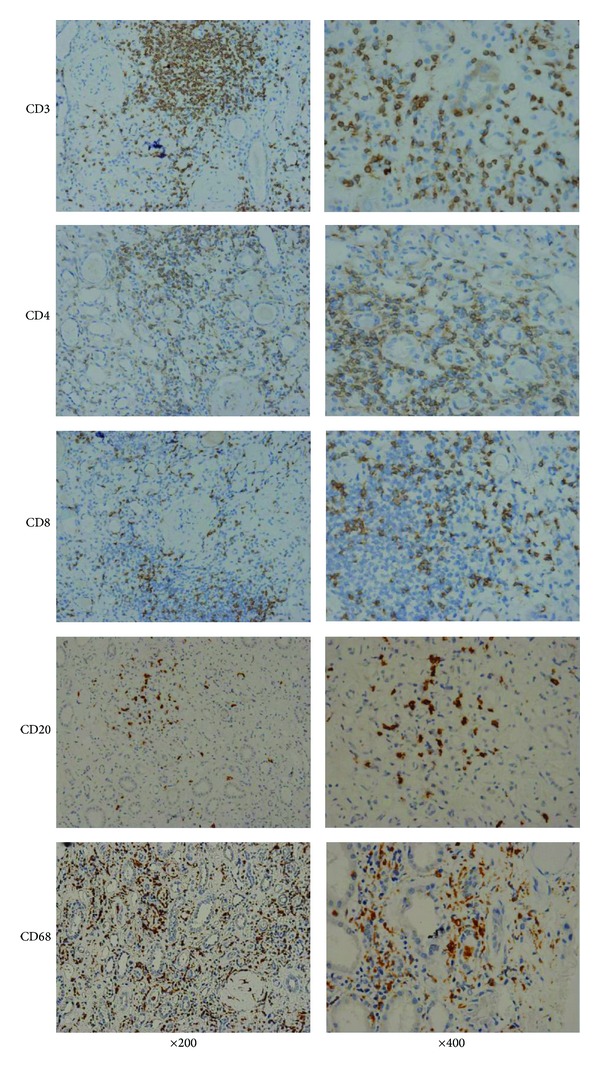
Immunohistochemical characterization of the inflammatory infiltrate in BK virus allograft nephropathy.

Formalin-fixed, paraffin-embedded renal biopsy sections were deparaffinized in xylene and rehydrated in graded ethanol (100%–70%). After deparaffinization, the sections were incubated with 3% H_2_O_2_ for 10 min to inactivate endogenous peroxidase. Microwave antigen retrieval was performed with citric acid solution (pH = 6.0) for 10 min. The slides were incubated with antibodies at room temperature for 1 hour. CD3, CD4, CD8, CD68, and CD20 were regularly detected (Figure 2). The antibody regimens were conducted as follows: mouse monoclonal antibodies against CD3 (1 : 100, CD3-PS1-S, Novocastra, Newcastle upon Tyne, UK), CD4 (1 : 50, NCL-CD4-1F6, Novocastra), CD8 (1 : 100, NCL-CD8-295; Novocastra), CD68 (KP1; Dako, Carpinteria, CA), and CD20 (1 : 500, L26; Dako). A single pathologist performed the blinded assessment of immunohistochemical data using a previously established quantitative immunostaining scoring method [15–17]. The immunostaining scoring method for CD3, CD4, CD8, CD20, and CD68 was performed as follows: 16 high-power fields were selected, and the amount of each type of mononuclear cell was calculated. As CD4, CD8, CD20, and CD68 cells take the overwhelming majority of infiltrating cells, the populations of CD4, CD8, CD20, and CD68 cells were added together for the density of total mononuclear cells (per mm^2^). Tubular HLA-DR staining was evaluated by visually assessing the approximate proportion of tubules. Tubule cell expression of HLA-DR was thought to be positive when stained cells represent ≥10% of the biopsy.

### 2.4. Statistical Methods

Statistical analyses were conducted using SPSS (v16.0) software. Pairwise comparisons of variables based on proportions were done by Fisher's exact test with Bonferroni correction for *P* value. Continuous variables were presented as mean ± SD and compared using one-way analysis of variance (ANOVA) followed by post hoc pairwise comparisons using LSD tests or analyzed using nonparametric method if the data were not normally distributed. Ordered categorical data were presented as median (25th–75th percentiles) and compared using the nonparametric Kruskal-Wallis ANOVA on ranks for global comparison, followed by Duncan's analysis for multiple comparisons. Spearman's correlation was used for analysis correlation. The level of statistical significance was set at *P* ≤ 0.05 (two-sided). 

## 3. Results

### 3.1. Baseline Patient Characteristics

Sixty-five renal allograft recipients were included in this study, including 22 cases of BKVN, 31 cases of AR, and 12 cases of stable allograft as controls. The baseline patient characteristics are listed in Table 1. None of the recipients had previously received an organ transplant. There were no significant differences among the three groups with respect to patient age, gender, time of prior transplantation, time of biopsy, or incidence of positive panel-reactive antibody. Each patient received anti-IL-2 receptor monoclonal antibody for the induction of immunosuppressive therapy and was subsequently maintained on a similar immunosuppressive protocol after transplantation (Table 1). 31 AR patients were classified as Banff I or II according to the Banff 97 criteria: 10 in the Banff I group and 21 in the Banff II group. Mycophenolic acid (MPA) area under the concentration curve from 0 to 12 hours (AUC_0–12_) and tacrolimus (TAC) 12 hr trough levels were routinely tested just before the biopsy, and patients with BKVN had significantly higher MPA AUC_0–12_ levels compared with those in the AR group and stable graft function group (45.68 ± 17.09 versus 29.90 ± 8.04 and 35.09 ± 7.89 mg·h/L, resp., *P* < 0.001). However, there were no significant differences in TAC levels compared with those in the AR group and stable graft function group (6.44 ± 2.27 versus 6.17 ± 2.65 and 6.95 ± 1.98 ng/mL, resp., *P* = 0.410).

### 3.2. Inflammatory Cellular Constituents in Different Groups

The morphological findings were quite similar between BKVN and AR. Tubulointerstitial nephritis with varying degrees of inflammatory infiltrates was a major histologic changes in both entities. A spectrum of viral inclusions could be identified in the tubules in 16 of 22 BKVN patients. We used immunohistochemistry to detect CD3, CD4, CD8, CD68, and CD20 expression. The number of lymphocytes positive by immunostaining for CD3, CD4, CD8, CD20, and CD68 for patients with BKVN and AR is shown in Table 2. In the BKVN group, the average values for CD3, CD4, CD8, and CD68 were similar to those in the AR group. However, the number of CD20-positive cells in renal biopsies of patients with BKVN and AR was 322 (range, 108–636) and 76 (range, 30–300), respectively, higher in the BKVN group (*P* < 0.001). Furthermore, when compared with stable allograft function group, every kind of lymphocytes is significantly higher in both BKVN and AR group. 

### 3.3. Proportions of Infiltrating Lymphocytes in BKVN and AR

The percentage of lymphocytes positive by immunostaining for CD3, CD4, CD8, CD20, and CD68 for patients with BKVN and AR is shown in Table 3. In BKVN, the values for the percentage of CD3, CD4, CD8, and CD20 were 37.6 ± 7.8, 20.5 ± 5.0, 18.4 ± 4.4, and 16.9 ± 12.6, respectively, and the corresponding values for AR were 51.2 ± 16.1, 27.3 ± 11.0, 23.9 ± 8.0, and 7.3 ± 9.2. The CD3 (*P* < 0.001), CD4 (*P* = 0.004), CD8 (*P* = 0.005), and CD20 (*P* = 0.002) values were all significantly different between the two groups (Table 3). However, there was no significant difference in the values for the percentage of CD68 between the two groups (45.5 ± 12.4 versus 41.4 ± 16.0, *P* = 0.324). To distinct BKVN from AR, we further compared the value of CD3/CD68 and CD3/CD20 between the two groups. There was no statistical difference in the value of CD3/CD68 (*P* = 0.059). However, the value of CD3/CD20 in BKVN group is significantly lower than that in AR group (3.88 ± 3.79 versus 12.76 ± 7.96, *P* < 0.001), and CD3/CD20 >5 and CD3/CD20 >10 may be of use to distinguish BKVN from AR (5/22 versus 25/31, *P* < 0.001, and 2/22 versus 15/31, *P* = 0.003, resp.).

### 3.4. HLA-DR Expression in BKVN and AR

Increased HLA-DR expression was observed in the tubule cells of 33 of the 53 renal transplants. HLA-DR expression was noted in 11/22 (50.0%) of the BKVN cases and 22/31 (71.0%) of the AR cases (Table 3). However, there was no statistically significant difference in tubule cell HLA-DR expression between the two groups (*P* = 0.156). According to the expression of HLA-DR, we divided the BKVN patients into two groups: HLA-DR-positive group and HLA-DR-negative group. The proportions of infiltrating cells (including CD3, CD4, CD8, CD20, and CD68) and the values of CD3/CD20 were not significantly different between the two groups (Table 4).

## 4. Discussion

This study revealed that immunophenotyping would aid in differentiating BKVN from acute rejection. We for the first time found that the percentages of CD3, CD4, CD8, and CD20 cells were all significantly different between BKVN and AR. In contrast, the presence of HLA-DR upregulation, however, may not be specific for acute rejection, since it may also be a response to BKVN. Furthermore, our study also found that the high MPA level appears to promote the development of BKV disease.

BKV infection is common after renal transplant, leading to BKVN, which is increasingly an important cause of graft failure. BKVN is a marker for an overimmunosuppressive state, and a reduction in immunosuppressive agents alone is a safe and effective therapy [18–22]. In contrast, patients with BKVN had rapid progression toward graft failure when they were treated with an antithymocyte agent or pulse steroids for presumptive acute rejection [23]. For diagnostic and clinical reasons, it is essential to carefully distinguish BKVN from AR. Immunophenotyping has gained widespread consideration for diagnosis and management of viral infections in immunocompromised hosts [8–11]. In this study, by comparing the interstitial lymphocytic infiltrates and proportions of infiltrating cells between patients with BKVN and AR, we found that immunophenotyping of the infiltrate would aid this distinction between the two entities.

In a small sample research, Ahuja et al. characterized the type of infiltrating lymphocytes in renal histology by immunophenotyping and found that the predominance of CD20-positive lymphocytes is suggestive of BKV infection [12]. In the current study, we also found the marked increase in the CD20 cells. On the other hand, in comparison with stable allograft function group, we confirmed in our samples that every kind of lymphocytes is significantly higher in both BKVN and AR group.

The proportions of infiltrating cells of BKVN have not been characterized previously. In comparison to patients with acute rejection, the percentages of CD3, CD4, and CD8 were all significantly fewer in BKVN, while CD20 took a much higher percentage. Furthermore, we compared the value of CD3/CD20 between the two groups and found that the value of CD3/CD20 in BKVN group is significantly lower than in AR group. And interestingly, we found that CD3/CD20 <5 and CD3/CD20 <10 are strongly correlated with BKVN compared with acute rejection. Differentiating BKVN from acute rejection is crucial in clinical practice, but, unfortunately, there is currently no test pathognomonic for BKVN. Histological diagnosis represents the gold diagnostic standard until now; however, it can be mistaken for allograft rejection, that is, tubulointerstitial nephritis with varying degrees of inflammatory infiltrates, tubulitis and tubular atrophy, and fibrosis. Immunohistochemistry with SV40 staining is now routinely used to document the presence of BKV in renal tissue; however, while renal involvement can be focal in earlier stages and could have predominant fibrotic changes with minimal inflammatory changes in the later stages of the disease [24], the results may represent false-negative biopsy results secondary to sampling error. Therefore, since negative biopsy results cannot rule out BKVN with certainty, CD3/CD20 < 5 and CD3/CD20 < 10 can be used as diagnostic algorithms for screening and monitoring “presumptive BKVN” whose renal allograft dysfunction may be associated with BK viremia and should be considered for renal transplant recipients who are not responding to antirejection treatment.

In this study, we found no statistically significant difference in tubule cell HLA-DR expression between BKVN and acute rejection. It is generally believed that tubular epithelial cells may show markedly increased HLA-DR expression during allograft rejection [25, 26]. However, there are arguments on the specificity of HLA-DR for acute rejection in BKVN patients. To clarify this issue, we divided the BKVN patients into two groups (HLA-DR-positive group and HLA-DR-negative group) according to the expression of HLA-DR. We found that the proportions of infiltrating cells (including CD3, CD4, CD8, CD20, and CD68) and the value of CD3/CD20 were not significantly different between the two groups. Therefore, we inclined to believe that the expression of HLA-DR may be stimulated by BK virus. These findings are consistent with the theory that the presence of HLA-DR upregulation may not be specific for acute rejection, and it may also be a response to influx of inflammatory cells secondary to BKV-induced parenchymal injury [27]. And this theory corresponds with the fact that treatment with corticosteroids did not improve renal allograft function [28]. Therefore, a definitive diagnosis of rejection concurrent with viral nephropathy should only be made if there is endarteritis, fibrinoid arterial necrosis, glomerulitis, or accumulation of the complement degradation product C4d along peritubular capillaries [29, 30]. 

In addition, we found that patients with BKVN had significantly higher MPA AUC_0–12_ levels at the time of diagnosis compared with matched controls, while TAC levels showed no significant differences among the three groups. Nearly all experts believe that the degree of immunosuppression is the primary risk factor for development of BKVN and that the reduction of immunosuppression is the principal treatment of BKVN [1, 4, 5, 29, 31–36]. Nevertheless, the specific role of different immunosuppressive agents as risk factors for BKVN is far from elucidated. Some studies found that the use of mycophenolate mofetil appears to promote the development of BKV disease [37, 38], while others believed that TAC level and prednisone daily dose other than mycophenolate mofetil (MMF) dose were associated with BKVN [39]. However, the association of MPA AUC_0–12_ level, and BKVN has not yet been evaluated. In our transplant center, we only monitor TAC 12 hr trough levels for adjustment of the dose during the followup like most transplant centers yet the MPA AUC_0–12_ levels were only routinely tested just before the biopsy, leading to the similar values for TAC levels of patients in different groups. This observation suggests that BKVN is associated with MPA AUC_0–12_ level and early detection of MPA level may be of value in avoiding the development of BKVN.

In summary, the findings in this study indicate that due to the focal nature of BKVN, a negative biopsy cannot rule out the disease. However, evaluation of a renal biopsy in combination with the immunophenotyping is necessary for the accurate determination of BKVN. On the other hand, the presence of HLA-DR upregulation may not only be specific for acute rejection but also be a response to BKVN. Moreover, this observation suggests that BKVN is associated with MPA AUC_0–12_ level, and early detection of MPA level may be of value in avoiding the development of BKVN.

## Figures and Tables

**Table 1:** Clinical characteristics of patients that participated in this study.

Characteristics	BKVN (*n* = 22)	AR (*n* = 31)	SF (*n* = 12)	*P* value
Gender, male (%)	16 (72.73)	19 (61.29)	8 (66.67)	0.686
Age (years)	36.31 ± 10.39	39.10 ± 6.89	40.08 ± 9.26	0.527
Donor age (years)	42.35 ± 7.67	46.65 ± 9.54	46.63 ± 7.96	0.349
Banff 97 (IA : IB : IIA : IIB)	—	4 : 6 : 11 : 10	—	—
Positive pretransplant PRA (*n*)	0	0	0	—
Previous transplant	0	0	0	—
Cold ischemic time (h)	8.14 ± 1.04	7.69 ± 1.16	7.92 ± 0.90	0.473
Warm ischemic time (min)	6.71 ± 1.67	6.35 ± 1.34	6.76 ± 1.26	0.724
Induction with IL-2R antibody, *n* (%)	22 (100)	31 (100)	12 (100)	—
Baseline immunosuppressants				0.118
MMF + Tac + Pred	21	23	9	
MMF + CsA + Pred	1	8	3	
MPA AUC_0–12_ (mg·h/L)	45.68 ± 17.09	29.90 ± 8.04	35.09 ± 7.89	<0.001
Tac level (ng/mL)	6.44 ± 2.27	6.17 ± 2.65	6.95 ± 1.98	0.410
Time of biopsy after Tx (month)	14 (10–21)	15 (2–25)	6 (2–16)	0.10

BKVN: BK virus nephropathy; AR: acute rejection; SF: stable allograft function; PRA: panel-reactive antibody; IL: interleukin; MMF: mycophenolate mofetil; Pred: prednisolone; Tac: Tacrolimus; CsA: cyclosporine A; MPA: mycophenolic acid; AUC_0–12_: area under the concentration curve from 0 to 12 hours; Tx: transplantation.

**Table 2:** Inflammatory cellular constituents in different groups.

	BKVN (*n* = 22)	AR (*n* = 31)	SF (*n* = 12)	Post hoc		
				*P*_12_	*P*_13_	*P*_23_
CD3 (cells/mm^2^)	502 (402–753)	624 (414–773)	180 (122–267)	0.958	<0.001	<0.001
CD4 (cells/mm^2^)	272 (177–389)	294 (207–388)	98 (69–147)	0.743	<0.001	<0.001
CD8 (cells/mm^2^)	276 (184–400)	278 (196–436)	96 (45–129)	0.770	<0.001	<0.001
CD20 (cells/mm^2^)	322 (108–636)	76 (30–300)	14 (11–23)	<0.001	<0.001	0.003
CD68 (cells/mm^2^)	646 (250–858)	480 (259–617)	85 (25–138)	0.055	<0.001	<0.001

BKVN: BK virus nephropathy; AR: acute rejection; SF: stable allograft function.

*P*_12_ means *P* value for BKVN group and AR group, *P*_13_ means *P* value for BKVN group and SF group, and *P*_23_ means *P* value for AR group and SF group.

**Table 3:** Proportions of infiltrating lymphocytes and HLA-DR expression in BKVN and AR.

	BKVN (*n* = 22)	AR (*n* = 31)	*P* value
CD3/total (%)	37.6 ± 7.8	51.2 ± 16.1	<0.001
CD4/total (%)	20.5 ± 5.0	27.3 ± 11.0	0.004
CD8/total (%)	18.4 ± 4.4	23.9 ± 8.0	0.005
CD20/total (%)	16.9 ± 12.6	7.3 ± 9.2	0.002
CD68/total (%)	45.5 ± 12.4	41.4 ± 16.0	0.324
CD3/CD68	0.97 ± 0.61	1.84 ± 2.36	0.059
CD3/CD20	3.88 ± 3.79	12.76 ± 7.96	<0.001
CD3/CD20 > 5	5	25	<0.001
CD3/CD20 > 10	2	15	0.003
HLA-DR ≥ 10%	11	22	0.156

BKVN: BK virus nephropathy; AR: acute rejection; SF: stable allograft function; HLA: human lymphocyte antigen.

**Table 4:** Proportions of infiltrating lymphocytes in HLA-DR-positive and HLA-DR-negative BKVN patients.

	HLA-DR < 10% (*n* = 11)	HLA-DR ≥ 10% (*n* = 11)	*P* value
CD3/total (%)	36.8 (30.7–43.1)	35.2 (30.5–44.8)	0.847
CD4/total (%)	19.9 (17.7–22.4)	19.9 (16.4–24.9)	0.652
CD8/total (%)	17.3 (13.8–23.1)	18.6 (13.7–22.4)	0.699
CD20/total (%)	13.1 (4.2–17.9)	16.2 (11.5–25.8)	0.217
CD68/total (%)	52.4 (35.5–55.8)	45.2 (39.6–49.0)	0.309
CD3/CD68	0.76 (0.60–1.09)	0.75 (0.63–1.00)	0.844
CD3/CD20	3.23 (1.61–9.70)	2.16 (1.48–3.56)	0.178

BKVN: BK virus nephropathy; HLA: human lymphocyte antigen.

## Abbreviations

BKVN: BK virus nephropathy
AR: Acute rejection
SF: Stable allograft function
HLA: Human lymphocyte antigen.
